# Engineering, global health, and inclusive innovation: focus on partnership, system strengthening, and local impact for SDGs

**DOI:** 10.3402/gha.v9.30175

**Published:** 2016-01-19

**Authors:** Katie L. Clifford, Muhammad H. Zaman

**Affiliations:** 1 Howard Hughes Medical Institute, Chevy Chase, MD, USA; 2 Department of Biomedical Engineering, Boston University, Boston, MA, USA

**Keywords:** Sustainable Development Goals, STEM, higher education, international partnerships, engineering research, global health technology

## Abstract

The recent drafting of the Sustainable Development Goals challenges the research community to rethink the traditional approach to global health and provides the opportunity for science, technology, engineering, and mathematical (STEM) disciplines, particularly engineering, to demonstrate their benefit to the field. Higher education offers a platform for engineering to intersect with global health research through interdisciplinary partnerships among international universities that provide excellence in education, attract nontraditional STEM students, and foster a sense of innovation. However, a traditional lack of engineering–global health collaborations, as well as limited faculty and inadequate STEM research funding in low-income countries, has stifled progress. Still, the impact of higher education on development efforts holds great potential. This value will be realized in low-income countries through strengthening local capacity, supporting innovation through educational initiatives, and encouraging the inclusion of women and minorities in STEM programs. Current international university-level partnerships are working towards integrating engineering into global health research and strengthening STEM innovation among universities in low-income countries, but more can be done. Global health research informs sustainable development, and through integrating engineering into research efforts through university partnerships, we can accelerate progress and work towards a healthier future for all.

## Introduction

On 25 September 2015, the United Nations General Assembly convened to officially adopt the Sustainable Development Goals (SDGs), an agenda that will guide development efforts for the next 15 years. One notable difference among this new set of goals is that the three Millennium Development Goals concerning health have been condensed into one SDG, declaring that development efforts should ‘ensure healthy lives and promote wellbeing for all at all ages’ (1). The summation of all health-related objectives into one overarching SDG should not be seen as lessening the importance of global health on sustainable development. Rather, this bold goal should challenge researchers and practitioners to embark on innovative, multidisciplinary approaches to creating robust technical capacity and effective and efficient public health solutions.

As UN Secretary-General Kofi Annan stated in a 2004 *Science* editorial, no nation ‘can afford to be without its own independent capacity in S&T’ (2). The international community has long recognized the importance of technology for sustainable development, but has not yet taken full advantage of the benefits engineering offers global health. From the development of the HIV rapid test, a point-of-care lateral flow assay that has reduced diagnosis time from days to minutes, to the creation of mobile drug verification devices that identify counterfeits through immediate on-site analysis, biomedical engineering has proven its impact on the delivery of health services worldwide. Engineering has the ability to bring disruptive technology into the realm of global health, providing solutions that complement the intensive work done by public health professionals, in turn accelerating the progress of global health initiatives and sustainable development.

## Barriers and potential solutions to inclusion of engineering in global health research

Whereas the value of engineering can be directly appreciated through the development of high-impact medical devices, the systematic inclusion of engineering in global health research, to date, has been largely limited. Traditionally, global health has been viewed purely as an extension of public health, with research primarily conducted in public health ‘silos’, stifling interdisciplinary collaboration. Likewise, engineers have not delved deeply into global health, as public health challenges have rarely been framed as technical questions. In addition to a lack of cross-disciplinary collaboration, fledgling biomedical engineering programs at universities in low-income countries are not sufficiently funded. Furthermore, local human capacity is limited in low-income countries, where few in-demand engineers are willing to leave their private sector or government jobs, often abroad, to accept faculty positions (3).

In order to ensure the sustainability of global health efforts, an element critical to the SDG, steps can be taken to strengthen engineering education and prepare a generation of public health problem-solvers. Global health research and development can greatly benefit from international university-based partnerships focused on providing an inclusive, interdisciplinary engineering education that fosters innovation in the local public health context. Partnerships with international universities can encourage a multidisciplinary approach to global health that embraces science, technology, engineering, and mathematical (STEM) solutions and accelerates the establishment of engineering research programs for low-income partners. As engineering programs grow, funding should follow suit, whether from private universities, foundations, international partnerships, or the government. Partnerships can also help low-income countries modernize their curriculum and encourage the inclusion of nontraditional STEM students, partly through collaboration among international students. Through initiatives supporting engineering in higher education, low-income countries can strengthen local capacity, building an engineering workforce knowledgeable in local public health and well-trained for global health engineering careers.

## State of engineering research in African universities: lagging growth in STEM fields leaves untapped potential

In order to ‘ensure healthy lives and promote well-being for all at all ages’ in accordance with the SDGs, low-income countries must develop an independent capacity for health care innovation and biomedical research at the university level. Higher education in many African countries has been described as ‘vastly underutilized’ (4) in development, particularly in the areas of science and technological innovation. Universities are critical in building engineering capacity in developing countries and are currently being ignored. Higher education plays a major role in research and development, generating unbiased information to inform policy and training the next generation of engineers and innovators. In 2009, it was estimated that only 2.1% of the world's researchers were based in Africa, down from 2.2% in 2002 (5). Furthermore, researchers in Africa only produced 2.0% of global publications in 2009, with a disproportionate number of those publications coming out of South Africa, Gabon, and Egypt (5). Currently, high-quality global health research that takes an interdisciplinary and inclusive approach is lacking at many higher education institutions in the developing world. This is particularly true for research at the intersection of engineering and global health.

Yet, there are a few partnerships between US universities and universities in low-income countries that serve as an example of how this model can be successfully employed to advance locally relevant public health technologies and strengthen local engineering capacity. In 2008, the Center for Innovation in Global Health Technologies at Northwestern University (Chicago, IL, USA) partnered with the University of Cape Town (Cape Town, South Africa) and the provincial Department of Health (Cape Town, South Africa) to design and create a digital radiography system (6). This system was created in response to a lack of available x-ray technology, partly due to the hefty recurring costs of film and development chemicals. After thorough evaluation of the facility and its patient burden, the universities jointly designed and manufactured a radiography system that met local needs while taking into account local resource limitations. The project has also employed the Kellogg School of Management at Northwestern University to perform a market analysis and investigate the economic impact of low-cost digital radiography on improved health outcomes (6).

The Institute of Biomedical Engineering at Oxford University (Oxford, UK), in partnership with the Indian Institute of Science (IISc) in Bangalore, is advancing affordable and practical prosthetics across India. Through funding from the Wellcome Trust, Oxford and the IISc will work together for 4 years to create prosthetics that are inexpensive and easy to maintain, yet suitable for the needs of the local population. The prototypic designs were created at the IISc, and the partners at Oxford will help guide those designs through the commercialization and clinical trials process (7).

Finally, Rice University, housing the most established global health engineering program in the United States, is collaborating with University of Malawi Polytechnic (Blantrye, Malawi) and the University of Malawi Medical School (Blantrye, Malawi) to design technologies that improve infant survival in low-resource settings (8). Through this collaboration, students developed a ‘bubble CPAP’ device, which has been shown to increase survival of neonates suffering from sepsis, low birth weight, or respiratory distress by 27% (9). This device is portable, durable, and costs 15 times less than infant CPAP machines currently on the market (9). In addition to the development of an impactful child health technology, the partnership focuses on bolstering local engineering capacity at the Polytechnic's engineering design studio. Through trainings offered in biomedical engineering and extracurricular hands-on educational opportunities, the collaboration focuses on inspiring innovative thinking among students from both institutions (8).

## Utilizing African universities in development: strengthening local capacity

The capacity for local independent innovation has not always been stressed, as can be seen by a lack of engineering and STEM educational opportunities in many developing countries. Often times, S&T has often been imported by developing countries from outside nations who did not consider local factors and regional needs when designing technologies (10, 11). The creation of a national science and technology agenda, one that includes higher education at the forefront of policy and translational research, will help drive economic growth and sustainable development for low-income nations (12).

One obstacle to global health research is the lack of local engineering capacity in the realms of research, teaching, and infrastructure. Due to inadequate funding, many professionals in the STEM fields do not go into teaching and research at local institutions, instead finding higher paying and more prestigious jobs abroad (3). Those who do decide to teach in higher academia are often underpaid and consequently are unmotivated to improve the curriculum and learning experience for their students (13). When it comes to research, oftentimes the projects that faculty take part in are not of local relevance, but instead are a means of obtaining personal promotion (5). Many academics focus heavily on consulting opportunities, leading to ‘de-institutionalization’ where teaching and research endeavors are pushed to the side in order to pursue more lucrative advising positions (14). These human resource challenges will only intensify as universities across Africa grow, with enrollment in sub-Saharan Africa increasing from 2.3 million in 1999 to 6.3 million in 2012 (5). As more students enter university, faculty workloads are increasing and more part-time professors are being hired to teach at a reduced overall cost (5). Without engaged and expert faculty who are financially and professionally supported by their institution, the creation of a cross-disciplinary global health and engineering research environment is unlikely.

Similarly, infrastructure required for health science research is not well funded, resulting in poorly maintained facilities and a lack of adequate research materials and equipment (2). In 2007, Africa's share of gross domestic expenditure on research and development globally was a minimal 0.9%, with no increase in investment from 2002 levels (15). Comparatively, many low-income countries and regions increased their share of gross domestic expenditure on R&D between 2002 and 2007: India's rose from 1.6 to 2.2%, Latin America's rose from 2.8 to 3.0%, and Asia's (new industrialized economies) rose from 5.1 to 6.3% (15). Lack of investment in research throughout Africa results in the degradation of university research facilities and the failure to provide students and researchers with the most up-to-date technologies.

## Utilizing African universities in development: innovation

Related to the lack of local capacity is the state of STEM education, which suffers from challenges of quality control and lack of emphasis on problem solving and innovative thinking. Presently, antiquated curricula, focused on rote memorization and lacking interdisciplinary integration, have hindered original research and innovation (13). There are limited experiential learning opportunities available to university students in Africa, as the higher education system does not promote a culture of internships. Furthermore, the curriculum and research focus in developing countries does not regularly prioritize local and national public health issues, failing to prepare university students in low-income countries for careers in local public health (13). Traditionally, health technology projects focus on device maintenance, training clinic staff and technologically savvy locals to troubleshoot technical problems and keep medical devices in working order. Although maintenance is very important in creating a sustainable health technology, it often overshadows the need to encourage *local* innovators to solve *local* problems within the *local* context. The UN Economic Commission for Africa, recognizing that creative thinking needs to be taught along with technical skills, created an initiative to advance biomedical engineering in teaching and research programs among African universities. The Engineering Expertise to Improve Health Outcomes in Africa initiative helps universities generate an engineering curriculum focused on innovation, as well as train technicians to build and maintain medical and laboratory equipment (16). The initiative further encourages entrepreneurial students through the International Medical Design Competition and the Biomedical Engineering Innovators School (16). By identifying talented students and pairing them with mentors who can guide them through their projects, as well as give valuable career advice, this initiative gets students involved in the STEM fields and helps create the future biomedical engineers that Africa desperately needs.

## Utilizing African universities in development: inclusion of nontraditional students

The health-focused SDG stresses the importance of health ‘for all’. Perhaps the best way to ensure the health of everyone, regardless of age, gender, race, or sexual orientation, is to encourage diversity in biomedical and health engineering education. International engineering-based collaborations should strive to include those students not traditionally found in engineering programs. On the whole, African universities have failed to attract women into science (3), so STEM programs should actively recruit women, minorities, and non-science students. One way to attract nontraditional students to engineering is through cross-concentration courses and programs with global health. Traditionally, global health attracts female and foreign students, so multidisciplinary programs can introduce engineering to a demographic of students who may not otherwise enter the field.

## International partnerships enhance the role of African universities in development

Traditionally, universities in the developing countries have not prioritized partnerships, failing to see the value in interdisciplinary collaborations and how partnering with international institutions can benefit their own STEM research agenda. Similarly, according to the International Association of Universities, few institutions in developed nations seek out academic partnerships with African universities, outside of simply philanthropic and development collaborations (17). Primarily, the partnerships forged are only to lend a helping hand to an African university and do not aim to advance the ‘excellence’ of the developed country partner in any way (14). Since it is often the institution in the developed country that is establishing and funding the partnership, these collaborations have often been defined solely by the agenda of the initiating partner, with little to no input from the partnering institution in the developing country (14).

Still, partnerships between developed country universities and African universities hold great promise, especially for advancing STEM research and education in the global health field. In our current globalized world, international partnership between universities are considered a critical part of successful research programs (14). University-level ‘north–south’ partnerships, between developed and developing countries, respectively, can and should benefit both entities. These type of partnerships can identify ‘southern’ students with talent in the STEM fields and provide an opportunity outside of their standard curriculum to explore engineering and its applicability to global health research. They can offer these students specialized training that their institution may not be able to provide and give them access to the resources and professional networks that ‘northern’ universities possess (18). Additionally, these partnerships can provide students from northern countries with alternative career choices in engineering and an opportunity to discover how their skills can be applied to global health in a real-world setting. Nevertheless, north–south university partnerships should be founded on a relationship of mutual respect and equal value, with a focus on eventual self-reliance (18). In addition to the benefits gained by the northern universities, a primary aim of the partnership should be to determine areas that need strengthening within the southern institution and to bolster teaching and research capacity based on locally identified needs.

The quality of the partnership should be regularly reevaluated, as both partners may not have the means and experience, especially for engineering research, to maintain high-quality facilities and instruction. Through collaboration, each institution is provided the opportunity to modify and revamp existing curriculum to align it with the multidisciplinary agenda of the partnership. Taking into consideration the pedagogical knowledge and experiences of their partnering institution, the joint creation of a modernized curriculum that meets the objectives outlined by both institutions for their students will strengthen the program and help institutions rethink their current teaching methods. The joint creation of a high-quality curriculum also allows for the inclusion of experiential learning opportunities. By integrating student-directed project-based learning into the program, participants will be able to learn from doing, with a hands-on approach that promotes problem-solving, teamwork, and real-world decision-making skills. Expanding on the idea of experiential learning, students gain access to instructors and researchers from both institutions, networking with experts in their field and receiving mentorship outside of what their individual institutions can provide. Whereas northern instructors may bring expertise in STEM and engineering disciplines to the partnership, southern instructors often provide global health expertise through a deeper understanding of local public health challenges and cultural context.

The physical sharing of facilities also benefits the partnership, specifically the partner that does not yet have an established STEM research program but is seeking to build one. STEM research, and particularly engineering programs, requires substantial investment for facilities, housing state-of-the-art equipment and supplies. Partnerships can provide the basis for a shared network of research facilities, mutually managed and open to joint research collaboration.

Whereas a quality partnership for engineering global health should feature inclusiveness, an excellence in education, and an interdisciplinary approach, it should also nurture an environment of innovation. Higher education in Africa was not designed to encourage outside-the-box thinking and foster innovation (4); instead the aim of a higher education has been to simply obtain a degree or certificate (13), with state-run research councils conducting most of the nationally relevant health research (4). However, the public health challenges of today require more creativity and resourcefulness than government research institutions are providing. Conversely, the university system in northern countries has traditionally been a center of innovation and free thought, a place where researchers were given rein to try new things. International partnerships focused on engineering global health can help spearhead creative solutions, with universities in the north bringing a long-held sense of innovation in higher education to institutions in the south. By envisioning the transition of the role of students from passive information recipients to active and inquisitive problem-solvers, northern universities can encourage students to ‘serve as agents of socioeconomic change rather than mere holders of degree certificates’ (13).

## Initial steps: the partnership for global health technology at Boston University

In order to realize the SDG of ensuring quality health care for all, low-income countries must improve their health delivery systems, particularly through strengthening research initiatives and encouraging local medical technology development. A critical step required for these improvements is strengthening STEM programming and fostering an innovative environment at the university level. The north–south university partnership model can accelerate the growth of a higher education system in low-resource countries that will educate the local medical innovators and public health leaders of tomorrow.

In an effort to pioneer this type of partnership, the College of Engineering at Boston University (BU) (Boston, MA, USA) has partnered with the State University of Zanzibar (SUZA) (Stone Town, Zanzibar) on a multi-year project to engineer a solution to a local health problem. Through funding provided by the Howard Hughes Medical Institute (Chevy Chase, MD, USA), the Partnership for Global Health Technologies connects university students from the United States and Tanzania who have a shared passion for improving health care in the developing world. In 2015, three undergraduates from BU spent their summer in Zanzibar, working directly with three SUZA medical students in developing a technological solution to a maternal health issue facing Zanzibar. During this time, the team conducted a comprehensive needs assessment to identify the technological gaps in maternal health care delivery at Mnazi Mmoja Hospital and the surrounding health facilities. Responses were collected from 172 individuals invested in quality maternal health care delivery, including physicians, nurses, lab technicians, traditional birth attendants, community health workers, and pregnant women seeking care. After analyzing survey responses, the team discovered that pregnancy-related hypertensive disorders were a leading complication in expectant mothers. One-quarter of women interviewed with pregnancy-related complications were found to have preeclampsia, and hypertensive complications were universally recognized by clinicians as a problem facing their patients.

Physicians, nurses, and lab technicians also noted the difficulties of monitoring renal and liver function in preeclamptic women, as severe hypertension and eclampsia can result in kidney and liver damage and even death. Currently, renal and liver function testing in Zanzibar must be performed in a lab, which is only open for testing during limited hours. Lab-based testing also requires reagents and supplies that often are out of stock, expired, or ineffective due to poor storage conditions. If renal or liver function testing is needed but cannot be provided by the facility, patients must pay for testing at a private hospital, which most cannot afford. Based on the results of the needs assessment and further consultation with local clinicians after analysis, the team has begun designing a point-of-care liver and kidney function diagnostic to be used for monitoring preeclampsia complications.

This student-driven program provides BU undergrads with the rare opportunity to apply their skills to solve real-world global health problems in the local context. At the same time, the program encourages innovative thinking, introduces SUZA students to engineering in the context of global health, and helps builds the local public health leadership of tomorrow. The program also makes a concerted effort to recruit nontraditional students to the engineering field, having accepted four female, three Muslim, and one African-American student during the first year.

## Conclusion

Over the last 15 years, the MDGs have directed efforts in global health research, but there is still much progress to be made. The SDGs will pick up where the MDGs left off, guiding us into a new era of global health research and encouraging us to approach sustainable global health development from a different direction. This approach should acknowledge the importance of STEM education in sustainable global health development and recognize the value of international university partnerships in accelerating engineering education in low-income countries. Successful partnerships – ones that foster innovation within the local context, encourage modernization of educational practices, share expert knowledge and resources, and promote inclusion among nontraditional STEM students – will help build local engineering capacity and advance medical device development. Through improvements in university-level STEM education and research, low-income countries can grow a sustainable workforce that possesses the engineering skills, innovative spirit, and public health passion required to take on the global health issues of our day.
